# Enlargements of the Liver.—II

**Published:** 1910-01-08

**Authors:** 


### Medicine.

### ENLARGEMENTS OF THE LIVER.—II.

Having discussed the physical signs of hepatic enlargement we may now consider the various causes of such enlargement; they may be classified in many different ways, but perhaps, for descriptive purposes, as good a subdivision as any is into smooth uniform enlargements on the one hand and local or irregular enlargements on the other.

### Causes of Enlargement of the Liver.

*A. Uniform, Smooth Enlargements.*—(1) Active congestion; (2) chronic congestion; (3) passive congestion; (4) fatty change; (5) catarrhal jaundice; (6) obstructive jaundice of other kinds, such as chronic pancreatitis, gall-stones, and so forth; (7) cirrhosis (unilobular stage); (8) rickets; (9) congenital syphilis; (10) lardaceous change; (11) leuchæmia; (12) Hodgkin's disease; (13) pernicious anæmia; (14) suppurative cholangitis; (15) suppurative pylephlebitis; (16) diffuse carcinoma; (17) diabetes mellitus; (18) phosphorus poisoning; (19) Weil's disease.

*B. Local and Irregular Englargements.*—(20) Carcinoma; (21) cirrhosis (multilobular); (22) perihepatitis; (23) gumma; (24) single abscess; (25) sarcoma; (26) hydatid cyst; (27) actinomycosis; (28) cystic degeneration.

A few words may be said about each of the above in turn:

### Active Congestion.

Active congestion is not common in Europe, but it is a frequent complaint in the tropics. It may occur as a complication of many of the acute fevers or malaria, or as a result of insufficient exercise associated with alcoholism and unwisdom at table. The liver becomes uniformly enlarged, the capsule is distended, and on section blood oozes freely from the cut surfaces. The viscus may be felt clinically to be slightly or moderately enlarged, and it is painful and tender when palpated.

The chief symptoms are pain and a feeling of fulness, weight, and oppression in the right hypochondriac region—sensations that are much increased by pressure—pain in the right shoulder, a bitter taste in the mouth, nausea, sickness, constipation, and scanty, high-coloured urine. The patient's temperature may rise to 102° F., and there may be difficulty in distinguishing the condition from hepatic abscess.

An estimation of the number of leucocytes may help to differentiate between these two conditions, a considerable increase in them favouring a diagnosis of suppuration. An examination of stained blood films may demonstrate the presence of malaria parasites if quinine has not been given, whilst even in the absence of the parasites a leucopenia, together with a relative increase in the large lymphocytes, would afford presumptive evidence in favour of malaria.

### Chronic Congestion (Tropical Enlargement).

This condition may follow active congestion, and it occurs chiefly in Europeans residing in the tropics. The liver is uniformly enlarged, its increase in size being either moderate or considerable. The viscus is usually painful and tender to pressure, the tenderness being generally most marked over the eighth and ninth right costal cartilages. The superficial epigastric veins may be dilated and there may be hæmorrhoids. The patient may be either thin and sallow or fat and flabby. The complexion is usually earthy or muddy, and the sclerotics tinged with yellow. The bowels are constipated, and the motions are apt to be pale.

### Passive Congestion (Nutmeg Liver).

A nutmeg liver is one that is uniformly enlarged as a result of over-distension of the intra-lobular veins with blood owing to mechanical obstruction to the return of the blood to the right auricle. The obstruction may be due to many different conditions, notably to failing compensation in cases of valvular heart disease, but also to failure of the right heart in cases of myocardial degeneration, adherent pericardium, arteriosclerosis, granular kidney, fibroid lung, emphysema, and so on; it may also arise when the heart itself is sound, but there is compression to the inferior vena cava by thrombosis, fibrosis, or new growth at or above the level at which the hepatic veins enter it.

The capsule of the liver becomes distended and shiny, and on section blood oozes freely from the cut surfaces. The hepatic tissue may be dark red or it may resemble the surface of a cut nutmeg if the obstruction has been of some standing. In some cases the liver may attain so considerable a size that inspection reveals some fulness of the right hypochondriac and epigastric regions, with perhaps visible pulsation in the latter. The edge of the liver can usually be felt with ease, hard, sharp, and well-defined; in advanced cases it comes below the level of the umbilicus, and it can often be traced obliquely across the abdomen towards the left hypochondrium. The surface of the liver is smooth and firm. Pulsation may sometimes be distinctly felt. The patient may complain of considerable tenderness when the surface and edge of the liver are being palpated. Roughly speaking, the more recently and the more rapidly the passive hepatic enlargement has taken place the greater will be both the pain and the tenderness. A large nutmeg liver of long standing may be neither painful nor tender.

Œdema of the legs and ascites may be present in some cases, especially in adults, as further results of the backward pressure. In children with cardiac disease, however, it is unusual to find ascites and considerable enlargement of the liver coincidently. When ascites is present it may be difficult to determine whether the liver is enlarged or not; if the abdominal wall is not too tense the liver may be felt on "dipping" for it. The symptoms are not as a rule distinctive. Patients do not generally complain of much pain, but they may be troubled with a feeling of fulness, weight, and oppression in the right hypochondrium and epigastrium. In advanced cases the complexion may be sallow, and there may be slight jaundice and marked lividity. The urine is scanty, high coloured, loaded with urates, and it may contain albumen. As a rule this form of enlargement is not difficult to diagnose, for the symptoms and signs of the cardiac or pulmonary condition to which it is due are usually well marked, and the discovery of such a cause should point to passive congestion as the reason for the enlargement.

(*To be continued.*)

